# Ether: An Antidote of Cocain and Stovain Poisoning

**Published:** 1910-06-15

**Authors:** J. E. Engstead

**Affiliations:** Grand Forks, N. D.


### ETHER: AN ANTIDOTE OF COCAIN AND STOVAIN POISONING.

J. E. ENGSTEAD, M.D., GRAND FORKS, N. D.

Directly after the introduction of cocain to the medical and dental profession, a number of accidents were reported, many of them fatal. The novelty of the drug and its effects were fascinating, and the want of experience and knowledge of its dangers, unrealized as they were by the majority of physicians, made it an extremely dangerous agent in other than the most careful hands. In common with most practitioners, I had my share of cases of cocain poisoning, though fortunately none proved fatal. By far the greater number of deaths occurred in the hands of dentists. The use of the drug was an almost daily occurrence in their practice, and they seemed especially prone to disregard its dangers.

About fifteen years ago I had as a next-door neighbor a dentist with a very large extraction practice, and it became a not uncommon occurrence for me to be called in hurriedly to help revive some patient. To my knowledge he had at least fifteen cases of cocain poisoning. Some were mild cases; many were profound and extreme. The symptoms were rapid and shallow respiration, feeble or absent pulse, with unconsciousness; in some cases the patients were difficult to revive. Nearly all exhibited extreme mental anxiety and restlessness.

In the first few cases I was called on to treat, strychnin and morphine in combination were used with marked benefit. But, as cases kept multiplying, I found the action of these drugs too slow, and I decided that there must be something to counteract the poison more rapidly when life was in extreme danger. It was necessary to find a remedy that could be administered at any time and be instantaneous in its action. I soon found ether to be the required drug. This was administered as ordinarily given to produce surgical narcosis. Ether stimulates the vasomotor system, is a tonic to the heart muscles, stimulates the action of the respiratory centers and of the brain and of the pneumogastric nerve, and increases the pulmonary circulation in the first stages. While cocain inhibits the action of the heart, especially on the right side, it has also a marked inhibitory action on the respiratory centers of the brain. Death may occur from feeble respiratory movements of the so-called Cheyne-Stokes type, or asphyxia.

To me ether has proved extremely valuable. It has saved what seemed hopeless cases. It stimulates the heart and the respiratory system almost instantly. The pulse becomes fuller at once and of normal tension. The marked mental excitement is allayed as the patient goes under the influence of the ether, and the effect of the poison rapidly disappears. The individual regains consciousness as soon as the effect of the small amount of ether has disappeared.

To get the best results, the anesthetic administered only to the degree of mild surgical narcosis, or, at times, even less than this. A mask should be employed and the ether given by the drop method. This is all-important. Given by the old method, the ether would only add to the danger of asphyxia by excluding air from the venous blood engorged lungs.

It occurred to me that this suggestion might be of help to others as it has been to me. Just at present there is much interest in the work of Jonnesco and his use of stovain. Many physicians will undoubtedly be led to try stovain, or cocain, or some other synthetical preparation whose action is similar. While in Paris some years ago it was my privilege to witness Tuffier perform a number of operationt under spinal anesthesia with cocain, all done in the brilliant style of this acknowledged master of surgical technic. In the last operation I saw in his clinic the patient did not bear the drug well, but began to show some very dangerous symptoms, and, in spite of the usual remedies, was soon *in extremis*. At my suggestion a few drops of ether were administered and all dangerous symptoms dissappeared immediately.—*The Journal, A. M. A.*, March 19th, 1910. P. 964.

